# Supplementary material for: Esculetin as a Bifunctional Antioxidant Prevents and Counteracts the Oxidative Stress and Neuronal Death Induced by Amyloid Protein in SH-SY5Y Cells
Source: Antioxidants (Basel). 2020 Jun 25;9(6):551. doi: 10.3390/antiox9060551 (PMC7346165; doi:10.3390/antiox9060551)
Supplement: Supplementary file 1 [file antioxidants-09-00551-s001.zip › Supplementary files/Figure S1.docx]

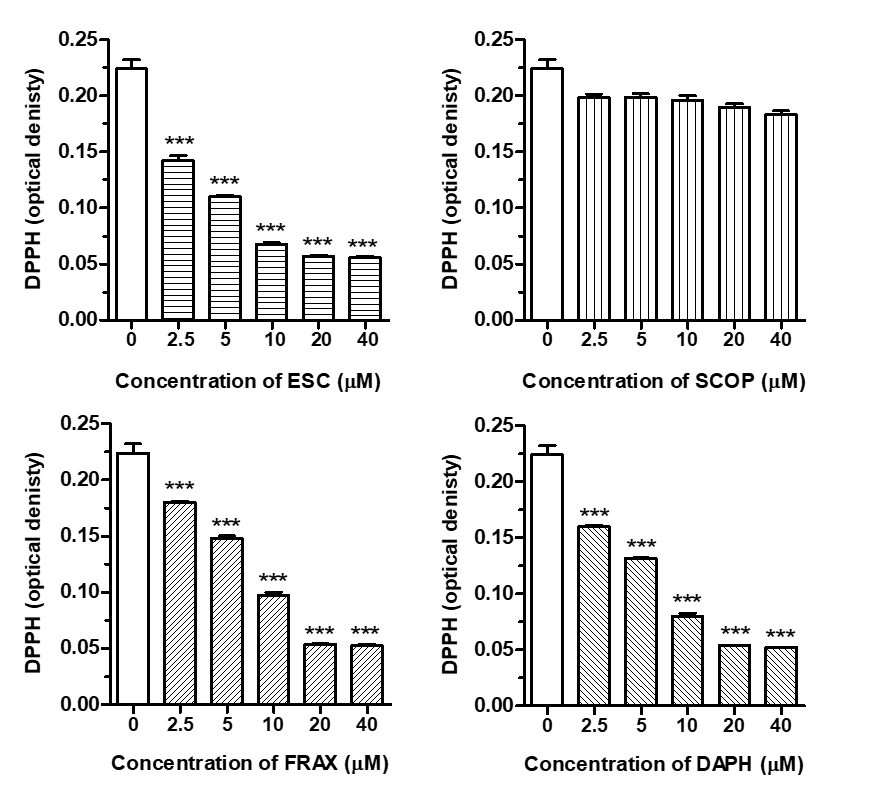


**Figure S1.** DPPH radical-scavenging activity of the studied coumarins. The DPPH radical -scavenging activity was performed using the DPPH radical**.** Data are expressed as optical density and reported as mean ± SD of three independent experiments (*** p<0.001 versus DPPH radical).
