# Supplementary material for: Esculetin as a Bifunctional Antioxidant Prevents and Counteracts the Oxidative Stress and Neuronal Death Induced by Amyloid Protein in SH-SY5Y Cells
Source: Antioxidants (Basel). 2020 Jun 25;9(6):551. doi: 10.3390/antiox9060551 (PMC7346165; doi:10.3390/antiox9060551)
Supplement: Supplementary file 1 [file antioxidants-09-00551-s001.zip › Supplementary files/Figure S2.docx]

**(A)**

**(B)**

**Figure S2.** ESC counteracts the early mitochondrial dysfunction induced by OAβ_1-42_ in SH-SY5Y cells. Cells were incubated with ESC [20 µM] and OAβ_1-42_ [10 µM] for 4 hours. At the end of incubation, extracellular Tween 20-insoluble MTT (TI-MTT) (A) and intracellular Tween 20-soluble MTT (TS-MTT) (B) were measured by using MTT formazan exocytosis assay as described in the materials and methods section. The levels of TS- and TI-MTT are expressed as percentages with control (untreated cells) set at 100 %. Data are reported as mean ± SD of at least three independent experiments (^§§§^p<0.001 versus untreated cells; ***p<0.001 versus cells treated with OAβ_1-42_ at one-way ANOVA with Bonferroni post hoc test).
